# Antipsychotic Drug Development: From Historical Evidence to Fresh Perspectives

**DOI:** 10.3389/fpsyt.2022.903156

**Published:** 2022-06-16

**Authors:** Katrina Weston-Green

**Affiliations:** ^1^Neurohorizons Laboratory, Molecular Horizons and School of Medical, Indigenous and Health Sciences, Faculty of Science, Medicine and Health, University of Wollongong, Wollongong, NSW, Australia; ^2^Illawarra Health and Medical Research Institute, Wollongong, NSW, Australia; ^3^Australian Centre for Cannabinoid Clinical and Research Excellence, New Lambton Heights, NSW, Australia

**Keywords:** schizophrenia, antipsychotic, treatment resistance, drug development, clinical trials, novel therapeutics, efficacy, side-effects

## Abstract

Schizophrenia is a complex disorder of varied etiology, manifesting symptoms that can differ between patients and change throughout an individual's lifespan. Antipsychotic drugs have evolved through first (e.g., haloperidol), second (olanzapine and clozapine) and a possible third (aripiprazole) generation of drugs in an attempt to improve efficacy and tolerability, with minimal side-effects. Despite robust scientific efforts over the past 70 years, there remains a need to develop drugs with greater efficacy, particularly in relation to the negative and cognitive symptoms of schizophrenia, addressing treatment resistance, with a lower side-effects profile compared to existing antipsychotic drugs. Identifying and investigating novel therapeutic targets remains an important component of future antipsychotic drug discovery; however, mounting evidence demonstrates neurobiological, neuroanatomical and functional heterogeneity in cohorts of individuals with schizophrenia. This presents an opportunity to refresh the approach to drug trials to a more targeted strategy. By increasing understanding of the basic science and pharmacological mechanisms underlying the potential antipsychotic efficacy of novel therapeutics prior to clinical trials, new drugs may be appropriately directed to a target population of schizophrenia subjects based on the drug mechanisms and correlating biological sub-groupings of patient characteristics. Improving the lives of sub-populations of people with schizophrenia that share common biological characteristics and are likely to be responsive to a particular compound may be more achievable than aiming to treat the complexities of schizophrenia as a homogenous disorder. This approach to clinical trials in antipsychotic research is discussed in the present review.

## Introduction: Schizophrenia Symptoms and Treatment

Schizophrenia is a chronic mental disorder of varied etiology, with diverse symptoms including retreat from reality, distorted thoughts, cognitive and motor impairment, emotional dysfunction and a decline in communication skills leading to social isolation, occupational disability and physical deterioration. It has been described as a conglomeration of syndromes rather than a single pathological state, due to the range of symptoms that can differ among individuals (1, 2). Broadly, schizophrenia consists of three symptom domains: positive, negative and cognitive. Positive symptoms include behavioral abnormalities such as speech and thought disorder, delusions and hallucinations, while the negative symptom domain encompasses a decline in response such as flattened emotional expression, alogia (lack of speech), avolition-apathy (emotional blunting) and anhedonia (inability to feel pleasure). The third symptom domain includes cognitive deficits, encompassing reduced executive function, such as organization, memory, learning and attention deficits, and altered perception e.g., misinterpretation of behavior and intent of others (1, 3, 4). Pharmacological intervention through the use of antipsychotic drugs remains a key component of schizophrenia treatment; however, existing medications cannot alleviate all symptoms, which can change throughout a person's life. Unfortunately, the search for the “ultimate” novel antipsychotic appears to be frustrated by the complexity of the disease and a percentage of patients remain unresponsive to antipsychotic treatment (5–7). In addition, pharmacological treatment involves a life-long adherence to drugs that are associated with an array of side-effects (8–19). Therefore, despite robust improvements in psychopharmacological therapy since the serendipitous discovery of the early first generation or “typical” antipsychotic compound chlorpromazine in 1951, more research is required to aid discovery of highly efficacious drugs with low adverse side-effects.

## Antipsychotic Drugs – A Historical Overview

The “psychopharmacological revolution” of the 1950-70's yielded the development of a number compounds, such as haloperidol, fluphenazine, loxapine and thioridazine, that were termed first generation “typical” antipsychotics. In 1966, a simple two page document suggested that because chlorpromazine and haloperidol were antagonists of amphetamine and that the hyperlocomotor activity effect of amphetamines was “…probably induced by the activation of dopamine receptors in the brain,” it was possible that dopamine receptor blockade was a mechanism of action of antipsychotic drugs (20). This led to the first biologically-based hypothesis on the etiopathology of schizophrenia (20); indeed the dopamine hypothesis has remained a prominent contender to-date (21). Subsequent research demonstrated a correlation between clinical potency and antipsychotic drug binding affinity to the dopamine D2 receptor sub-type, with occupancy of between 60 – 80% of striatal D2 receptors inducing a therapeutic response (22–24). D2 receptor blockade could theoretically dampen the post-synaptic response to a hyperdopaminergic state in the schizophrenia brain [see McCutcheon et al., (25)] for a review of dopamine in schizophrenia), but leads to motor and extrapyramidal side-effects (EPS) (e.g., Parkinsonism, acute dystonia, akathisia, tardive dyskinesia) at occupancy levels >78% (26, 27). These side-effects were considered to be an unavoidable component of antipsychotic treatment, but the concerning acute and long-term consequences of EPS in some patients warranted strict recommendations on the use of “neuroleptics” and called for further research into drug development (28). In addition, a significant portion of patients were unresponsive to first generation antipsychotics (5) even though the central uptake of a D2 antagonist ([^18^F]N-methylspiroperidol) was the same between resistant and responsive patients following haloperidol administration (29). This indicated that the absence of antipsychotic effect in non-responsive schizophrenia was not due to altered D2 receptor binding or drug uptake alone and that other factors may differ between patient groups. Thus, a need was identified for a new line of highly efficacious pharmacological therapeutics without the extrapyramidal effects of first generation antipsychotics.

In 1974, a report by Niskanen et al. (30) demonstrated the efficacy of clozapine (produced by Wander Pharmaceuticals, Switzerland, 1958) in treating a percentage of antipsychotic-unresponsive patients compared to chlorpromazine. However, it was rapidly removed from the market following associated fatal agranulocytosis (31). Despite the initial set-back, a large-scale, double-blind study (32) reported the efficacy and safety of clozapine in treating non-responsive schizophrenia (vs chlorpromazine), including lower EPS scores, when patients were closely monitored for blood abnormalities, assisting the launch of clozapine onto the USA market in 1990. Thus began the rapid development of a new “second generation” of antipsychotic drugs, such as olanzapine, clozapine, risperidone and amisulpride. In 2003, a meta-analyses incorporating 152 studies showed greater effect sizes with these particular drugs across multiple therapeutic parameters [Positive and Negative Syndrome Scale (PANSS), Brief Psychiatric Rating Scale (BPRS) and the Clinical Global Rating (CGI)] compared to first generation and several other second generation antipsychotics (33). Clozapine was particularly promising in the treatment of negative symptoms, with improved emotional withdrawal, blunted affect and anhedonia reported in people with non-deficit (negative symptom-dominant) schizophrenia (34, 35); however, accumulating evidence has revealed less consistency in efficacy findings across studies over the years (36). Unlike typical first generation antipsychotics that were primarily known as D2 receptor antagonists, atypical second generation antipsychotics affected a range of receptors (dopamine D1, D2, D3 and D4, adrenergic α_1_ and α_2_, histaminergic H_1_, muscarinic M_1_, M_3_ and M_4_, ionotropic NMDA and metabotropic glutamate, and serotonergic 5-HT_1A_, 5-HT_2A_, 5-HT_2C_, 5-HT_6_ and 5-HT_7_, with lower propensity to cause EPS than first generation drugs (16, 37–40). However, while a broad receptor binding profile could enable drug effects on multiple targets for broader symptom efficacy, it is also likely to underlie the numerous adverse side-effects associated with the use of second generation antipsychotic drugs, including metabolic side-effects (16, 19, 41–44). Therefore, while atypical antipsychotics had gained popularity for both approved and off-label prescription (45–48), the side-effects profile, persistence in symptoms in some individuals and, more recently, the question of whether these drugs were actually better than typical antipsychotics (49), remained important factors.

The more recent introduction of aripiprazole seemed to provide a different approach to schizophrenia treatment; acting largely on the dopamine D_2_ receptor (similar to first generation typical antipsychotic drugs) but with a lower risk of inducing EPS, it could treat broader symptoms of schizophrenia (similar to some second generation atypical antipsychotics) but had a lower metabolic liability (50). Several studies suggested that aripiprazole was a D2 partial agonist that exerts differential intrinsic activity on the D2 receptor depending on local dopamine levels (51, 52); i.e., activates D2 receptors in the absence of dopamine (though to a lesser extent than dopamine), but inhibits dopamine binding to the D2 receptor in the presence of dopamine (i.e., competitively binds to D2 receptors to decrease receptor activity) (51). The functional outcome of such a mechanism could be a reduction in D2 receptor activity in hyperdopaminergic regions of the schizophrenia brain (i.e., the mesolimbic pathway), while having mild effect on the nigrostrial pathway that would contribute to the reduced incidence of EPS with aripiprazole treatment (52–55). Furthermore, the broader 5-HT_1A_ partial agonist and 5-HT_2A_ antagonist properties of aripiprazole may contribute to its ability to treat the negative symptoms of schizophrenia in some patients (56). Therefore, aripiprazole and similar partial agonists, such as the more recently developed cariprazine and brexpiprazole (57, 58), could be considered a new class of antipsychotic drug – a possible third generation of antipsychotics (55). Aripiprazole has been considered efficacious with minimal side-effects (56). For example, Kane et al. (59) reported minimal efficacy differences between aripiprazole and haloperidol in individuals treated for acute relapse, e.g., 77:74% responders, respectively, with significantly higher tolerability and less discontinuation over a 1-year period (59). While this response rate is important, these findings showed that symptoms in 23–26% of acutely ill individuals in this study remained persistent (59).

## Treatment Resistance Despite +70 Years of Antipsychotic Drug Discovery

Despite continued developments into understanding the etiology of schizophrenia and the mechanisms of antipsychotic drug efficacy, there remains a 20–60% incidence of treatment resistance (60, 61). A patient is considered treatment resistant when significant improvements in symptoms are not apparent after administration of two different antipsychotic drug classes for at least 2–8 weeks of therapy (60). Clozapine is considered superior in treating previously resistant patients; however, significant improvements are only experienced by 30–50% of treatment-resistant patients and the side-effects of clozapine (discussed above) are important considerations (60, 62). Furthermore, existing antipsychotics mostly work on the positive symptoms of schizophrenia, with less long-term efficacy in treating the negative symptoms and generally minimal to no benefit on the cognitive domain of schizophrenia. Cognitive impairment has been recognized as a core feature of schizophrenia from which other symptom domains may arise (63) and affects 80% of patients (64–66). Large-scale studies, such as the Clinical Antipsychotic Trials of Intervention Effectiveness (CATIE) and meta-analyses, have highlighted minimal or no overall effect of antipsychotics on cognition (67, 68), while other clinical and rodent studies suggest that antipsychotics can worsen cognitive function, particularly typical 1^st^ generation drugs (17, 69, 70). Therefore, further development of compounds that can treat the multiple domains of schizophrenia, devoid of treatment-resistance and side-effects, is still required. A number of new neurotransmitter system targets are currently being investigated with the aim of discovering potential novel therapeutics [reviewed in (71)]; indeed, evidence including large-scale genome-wide association studies have identified hundreds of novel predicted gene targets for the treatment of schizophrenia, suggesting that multi-target approach may be required (72).

## Narrowing the Approach to Novel Treatments – Is it Appropriate to Seek *P* < 0.05 Treatment Efficacy vs. Controls?

While the discovery of novel therapeutic targets (or combinations of) are one part of the puzzle toward improving the treatment of schizophrenia, another important consideration is heterogeneity of the target population. The variable response of patients to first, second and a possible third generation of antipsychotic drugs, each with different mechanisms of action, inherently demonstrates a level of heterogeneity between individuals. Indeed, a number of studies demonstrate the existence of sub-types or biotypes within schizophrenia populations. For example, Tamminga et al. (73) proposed moving away from clinical phenomenology-based approaches in the diagnosis and discovery of molecular treatments, to a focus on neuroanatomical, and cellular and molecular characteristics of individuals. They described phenomenology (i.e., broadly defining and classifying symptoms based on observable behaviors) as “suboptimal for capturing neurobiological distinctiveness”. Utilizing the Bipolar and Schizophrenia Network for Intermediate Phenotypes, Tamminga et al. (73) proposed 3 biotype clusters of individuals that considered cognitive scores, electroencephalography (EEG) power, gray matter volume, incidence of affected relatives and cannabis use. The biotype with the lowest cognitive scores also exhibited lower gray matter volume, low EEG power and lowest cannabis use, while the biotype with nearly normal cognition and EEG power also had normal gray matter volume and high cannabis use (73). Along the same lines of heterogeneity, Dean and Scarr (74) demonstrated a sub-group of individuals with schizophrenia that exhibit 76% lower levels of cortical muscarinic M1 receptors ([(3)H]pirenzepine binding) and lower M1 receptor mRNA levels compared to controls (74, 75). This group accounted for 26% of the 80 schizophrenia subjects examined and was termed the muscarinic receptor-deficient schizophrenia (MRDS) subgroup (75). As another example, evidence suggests the existence of a subgroup of patients with an “elevated inflammatory biotype”; that is, a subgroup of people (~40% of the 43 individuals in the schizophrenia cohort) that exhibit increased peripheral pro-inflammatory cytokines and low cognitive performance (76–78). In addition, according to Potkin et al. (79) treatment resistance, amounting to approximately 36% of people with schizophrenia, can be associated with various dopaminergic states, i.e., either dopamine super-sensitivity involving upregulation of dopamine receptors following chronic antipsychotic D2 antagonism or treatment-resistant individuals with normal dopaminergic activity; the latter of which may present with hyperdopaminergia stemming from dysregulated upstream excitatory glutamatergic inputs. Therefore, it is reasonable to suggest the existence of treatment-resistant subtypes and that dopaminergic state could contribute to differential antipsychotic response. Overall, this evidence demonstrates several examples of heterogeneity in the molecular and cellular characteristics of schizophrenia, with sub-groups spanning approximately 26–40% of a given cohort. Unfortunately, consideration of sub-groups are rarely translated to drug trials. If only 26–40% of a population of people with schizophrenia responded to a novel drug in a clinical trial, would it be considered a statistically significant treatment attempt? Interestingly, recent statistical publications have questioned the use of the “p” value, revisiting the idea that the P value, introduced by Ronald Fischer in 1920, was not intended to be a definitive answer, rather an indicator of whether the hypothesis was worthy of further examination (80, 81). The authors suggest that asking the question of how much of an effect is present, rather than “is there an effect,” is more appropriate than the yes or no approach of *P* values, that often cannot be replicated (80, 81).

Moving forward, in light of the existence of sub-populations and heterogeneity in schizophrenia, developing a better understanding of the basic science and pharmacological mechanisms of potential novel therapeutics prior to initiating clinical trials may inform a more targeted approach to likely responders. For example, xanomeline, has shown antipsychotic properties, with reduced BPRS and PANSS scores, and a particularly notable response in improving cognition (verbal and short term memory) (82), which seems to be in line with the role of cholinergic signaling in cognitive function (83). However, as a muscarinic M1 receptor agonist, xanomeline may exert differential response in MRDS individuals that could comprise 26% of a cohort (74, 75). Cannabidiol (CBD) is a non-intoxicating compound from the cannabis plant examined as a potential novel antipsychotic drug, with mixed results in terms of efficacy. In one study, Boggs et al. (84) reported no improvement in PANSS or MATRICS Consensus Cognitive Battery scores following 6 weeks adjunct CBD (600 mg/day) in individuals with schizophrenia compared to controls (*n* = 18/group). The cohort comprised individuals stably treated with first or second (clozapine excluded) generation antipsychotic drugs or multiple antipsychotics (representing 50: 27.8: 11% and 55.5: 72.2: 38.9% of people in the CBD treatment and placebo arms, respectively) and 38.9% of both treatment groups were treated with anticholinergic medications (84); i.e., sizable percentages of the cohort were responsive to medications with different pharmacological mechanisms of action. In contrast, McGuire et al. (85) reported significantly improved PANSS positive scores and CGI scale, and improved Global Assessment of Functioning and Brief Assessment of Cognition in Schizophrenia scores that fell short of significance (*p* = 0.08 and *p* = 0.068, respectively) following adjunct CBD (1,000 mg/day) treatment. As mentioned above, the question of whether these results should be interpreted as ineffective given the original intent of the *p* value remains unanswered (80, 81). Interestingly, the cohort included stable patients predominantly treated with second generation antipsychotic drugs (64–67% of the CBD and placebo groups), 25–29% of both groups were stable with aripiprazole (a potential third generation antipsychotic) and only a small percentage (6–9%) on first generation antipsychotics (85), which could suggest a more homogenous population compared to Boggs et al. (84). Whether cohort heterogeneity could negate statistical effects is unknown, but may be worthy of further exploration with a larger or more targeted patient sample. For example, CBD is a dopamine D2 receptor partial agonist (86) (similar to aripiprazole) that may not be the most efficacious option as an adjunct or replacement therapy for patients who are stably responsive to first generation D2 receptor antagonists. On the other hand, CBD is neuroprotective, with pro-cognitive and anti-inflammatory effects (3, 87); therefore, CBD may be targeted toward individuals with an inflammatory subtype (76–78), and individuals with a low gray matter volume and very low cognitive scores of biotype 1 reported in Tamminga et al. (73). Pre-clinical evidence suggests that cannabidiol restores muscarinic M1/M4 receptor densities and choline acetyltransferase (ChAT) protein expression in the hippocampus and pre-frontal cortex of males in the poly I:C model of schizophrenia (88); results that could have relevance to the MRDS sub-type (74, 75).

## Discussion / Conclusion

Encouragingly, a recent clinical trial of adjunct estradiol therapy by Thomas et al. (89) reported two subgroups within their schizophrenia cohort; those who responded to adjunctive estradiol therapy through decreased PANSS response (approximately 77% of the cohort), and those who were considered non-responders (i.e., unchanged PANSS scores) predicted by serum endocrine markers, estadiol and follicle stimulating hormone (FSH) levels. Unfortunately, this approach has not been the norm to-date. Perhaps it is the case that antipsychotic drug discovery has progressed over the past 70 years; however, careful consideration of target populations and changes to the way we look at efficacy could be beneficial. Given the lifetime of devastating outcomes of schizophrenia that lasts a person's lifetime, often resulting in lowered life expectancy, improving the lives of even 25% of a cohort through a novel medication approach could be considered “significant” at face value. Further research into biotypes and biomarkers in schizophrenia, as well as basic science experiments to increase understanding of pharmacological mechanisms of novel compounds are needed in order to better facilitate accurately targeted clinical trials.

## Author Contributions

KW-G conceptualized, drafted, and edited the manuscript.

## Conflict of Interest

The author declares that the research was conducted in the absence of any commercial or financial relationships that could be construed as a potential conflict of interest.

## Publisher's Note

All claims expressed in this article are solely those of the authors and do not necessarily represent those of their affiliated organizations, or those of the publisher, the editors and the reviewers. Any product that may be evaluated in this article, or claim that may be made by its manufacturer, is not guaranteed or endorsed by the publisher.
